# Subcortical Dendritic Scaffolding in Autism Spectrum Disorder: A Testable ANK2–SCN2A–SHANK Framework

**DOI:** 10.3390/ijms27135979

**Published:** 2026-07-03

**Authors:** Sara Cacciato-Salcedo, Ana Belén Lao-Rodriguez, Marija M. Petrinovic, Manuel S. Malmierca

**Affiliations:** 1 Cognitive and Auditory Neuroscience Laboratory (CANELAB), Institute of Neuroscience of Castilla y León, University of Salamanca, 37007 Salamanca, Spain; scacciato@usal.es (S.C.-S.); anabelenlao@usal.es (A.B.L.-R.); 2 Department of Cell Biology and Pathology, University of Salamanca, 37007 Salamanca, Spain; 3 Institute for Biomedical Research of Salamanca (IBSAL), 37007 Salamanca, Spain; 4 Department of Forensic and Neurodevelopmental Sciences, Institute of Psychiatry, Psychology and Neuroscience, King’s College London, 16 De Crespigny Park, London SE5 8AF, UK; marija-magdalena.petrinovic@kcl.ac.uk; 5 Department of Neuroimaging, Institute of Psychiatry, Psychology and Neuroscience, King’s College London, De Crespigny Park, London SE5 8AF, UK; 6 Medical Research Council Centre for Neurodevelopmental Disorders, King’s College London, New Hunt’s House, London SE1 1UL, UK

**Keywords:** autism spectrum disorder, dendritic integration, ankyrin-B, NaV1.2, SHANK3, striatum, thalamus, amygdala, cortico-subcortical circuits, postsynaptic density

## Abstract

The autism spectrum disorder-associated *SCN2A*, *ANK2*, and *SHANK*-family genes encode molecularly distinct proteins that converge functionally on dendritic integration. Recent work established that ankyrin-B, encoded by *ANK2*, acts as an obligate dendritic scaffold for NaV1.2, encoded by *SCN2A*, in neocortical pyramidal neurons. Loss of this module mislocalizes dendritic NaV1.2, reduces dendritic Na^+^ influx, weakens backpropagating action potentials, and impairs synaptic maturation and long-term potentiation. *SHANK* proteins organize a complementary postsynaptic receptor scaffold within dendritic spines, coupling N-methyl-D-aspartate (NMDA), α-amino-3-hydroxy-5-methyl-4-isoxazolepropionic acid (AMPA), and metabotropic glutamate receptor (e.g., mGluR5) signaling to the actin cytoskeleton through layered PSD-95/GKAP/Homer interactions. Disruption of this scaffold can destabilize excitatory transmission, spine morphology, and plasticity. We propose that these dendritic shaft and spine-associated modules jointly regulate dendritic input–output gain and that their disruption may contribute to autism spectrum disorder by destabilizing, rather than uniformly shifting, excitatory integration across cortico-subcortical circuits relevant to sensory reactivity, behavioral flexibility, and social-valence processing. Here, we review the cortical evidence for this layered dendritic convergence and evaluate its potential relevance beyond the cortex. We assess the striatum, thalamus, and amygdala as subcortical sites where related dendritic scaffolding mechanisms may operate. The striatum provides the strongest current test case, with established roles for both NaV1.2 and *SHANK3* in medium spiny neuron physiology and corticostriatal connectivity. Thalamic and amygdalar extensions are supported mainly by *SHANK*-related circuit and channelopathy data but lack direct evidence for *ANK2*–*SCN2A* involvement. The framework is experimentally testable: conditional *Ank2* deletion in striatal, thalamic, and amygdalar cell types; dendritic Na^+^/Ca^2+^ imaging across *Scn2a*, *Ank2*, and *Shank3* models; adult rescue experiments; and genetic-interaction designs would determine whether ankyrin-B supports dendritic excitability beyond the cortex and whether these genes converge on, rather than merely parallel, dendritic input–output gain. Validation in human subcortical tissue would then establish whether this dendritic scaffolding logic represents a shared point of convergence through which genetically distinct autism spectrum disorder-risk variants alter circuit function.

## 1. Introduction

Autism spectrum disorder (hereafter, *autism*) is a neurodevelopmental condition characterized by persistent differences in social communication and social interaction across multiple contexts, together with restricted and repetitive patterns of behavior, interests, or activities, with features present in the early developmental period and associated with clinically meaningful functional impact or support needs [1,2]. Its strong heritability and pronounced genetic heterogeneity have made gene discovery dependent on high-throughput genomic approaches. Next-generation sequencing, particularly trio-based whole-exome and whole-genome sequencing in large cohorts, has been central to this progress because it enabled the systematic identification of rare *de novo* and inherited variants associated with autism and helped resolve a genetic architecture in which many genes each contribute to a fraction of cases [3,4,5,6]. These efforts have placed *SCN2A*, *ANK2*, and members of the *SHANK* family, particularly *SHANK3*, among the highest-confidence autism-associated genes and genes associated with related neurodevelopmental conditions, including intellectual developmental disability, epilepsy, and developmental delay [3,4]. These genes encode molecularly distinct proteins: the voltage-gated sodium channel NaV1.2 (*SCN2A*), the membrane–cytoskeleton scaffold ankyrin-B (*ANK2*), and postsynaptic density organizing scaffolds (*SHANK* family). Their phenotypic range is broader than autism alone; therefore, this review frames them as components of an autism-relevant neurodevelopmental mechanism rather than as an autism-specific pathway.

**Figure 1:**
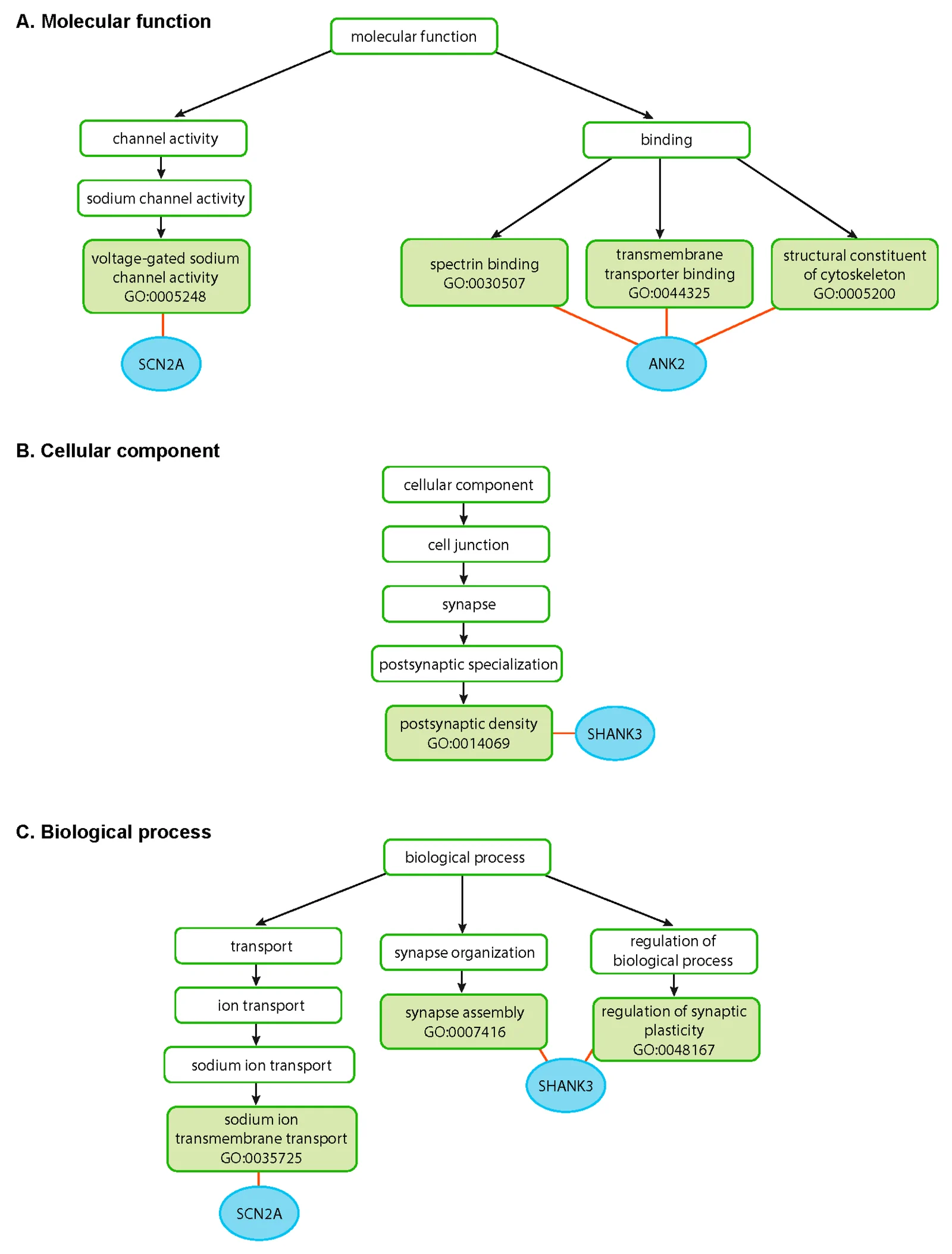
Gene Ontology overview of *SCN2A*, *ANK2*, and *SHANK3* functional annotations. Selected Gene Ontology (GO) annotations classify the encoded proteins according to (**A**) molecular function, (**B**) cellular component, and (**C**) biological process. *SCN2A*/NaV1.2 is represented by voltage-gated sodium channel activity and sodium ion transmembrane transport; *ANK2*/ankyrin-B by structural constituent of cytoskeleton, spectrin binding, and transmembrane transporter binding; and *SHANK3* by postsynaptic density, synapse assembly, and regulation of synaptic plasticity. Blue ellipses denote the genes; green-filled boxes, the selected focal GO terms; white boxes, broader ancestral GO terms; black arrows, *is_a* relationships; and red lines, gene-to-term annotations. The figure illustrates how molecularly distinct autism-risk genes converge on membrane-associated, cytoskeleton-linked, and postsynaptic mechanisms relevant to dendritic integration.

The biological functions of *SCN2A*, *ANK2*, and *SHANK3* can be contextualized through selected Gene Ontology (GO) annotations [7,8,9], which classify gene products according to molecular function, cellular component, and biological process (Table 1; Figure 1). *SCN2A* is associated with voltage-gated sodium channel activity and Na^+^ transmembrane transport, consistent with the role of NaV1.2 in neuronal excitability and sodium-dependent dendritic signaling. *ANK2* is associated with cytoskeletal structure, spectrin binding, and transmembrane transporter binding, consistent with the role of ankyrin-B as a membrane–cytoskeleton scaffold that couples membrane proteins to the spectrin–actin cytoskeleton. *SHANK3* is associated with the postsynaptic density, synapse assembly, and regulation of synaptic plasticity, consistent with its role as an excitatory postsynaptic scaffold. Together, these annotations support the central premise of this review: molecularly distinct autism-associated genes may converge on membrane-associated, cytoskeleton-linked, and postsynaptic mechanisms relevant to dendritic integration.

**Figure 2:**
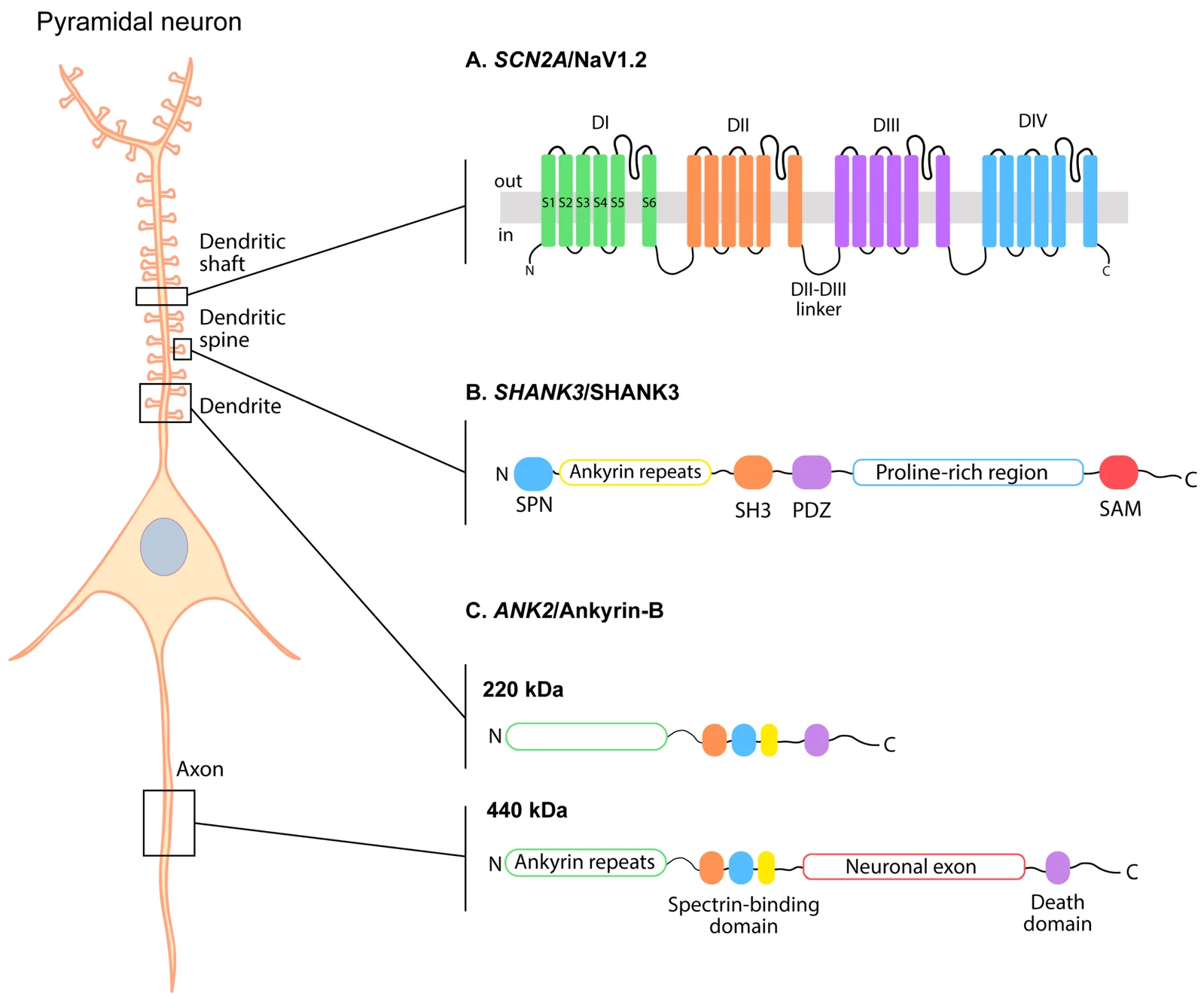
Protein domain organization of NaV1.2, ankyrin-B, and SHANK3 within a pyramidal neuron. The schematic neuron (**left**) depicts the principal subcellular compartments: dendritic shaft, dendritic spine, dendrite, and axon. Connecting lines link each compartment to the domain schematic of the protein or isoform enriched there. (**A**) NaV1.2 (*SCN2A*) is a pore-forming voltage-gated sodium-channel alpha subunit comprising four homologous transmembrane domains (DI–DIV), each with six membrane-spanning segments (S1–S6) oriented between the extracellular (“out”) and intracellular (“in”) sides of the membrane, with intracellular N- and C-termini and the ankyrin-binding motif located in the DII–DIII intracellular linker. (**B**) SHANK3 is shown with its SPN domain, ankyrin-repeat region, SH3 and PDZ domains, proline-rich region, and C-terminal SAM domain. Domain shapes and positions are schematic and not drawn to scale. (**C**) Ankyrin-B (*ANK2*) is shown as the canonical 220 kDa and giant 440 kDa isoforms, sharing the N-terminal membrane-binding ankyrin-repeat region, the spectrin-binding domain, and the C-terminal death domain (labels shown on the 440 kDa isoform; corresponding shapes are mirrored on the 220 kDa isoform); the neuronal giant exon is unique to the 440 kDa isoform.

This functional convergence is also supported by the domain organization of the encoded proteins (Figure 2). NaV1.2 is a pore-forming sodium-channel alpha subunit organized into four homologous transmembrane domains that support voltage sensing, pore formation, and fast inactivation [10,11]. Ankyrin-B contains membrane-binding ankyrin repeats, a spectrin-binding domain, a death domain, and a C-terminal regulatory region that allow it to connect membrane proteins to the spectrin–actin cytoskeleton [12]. SHANK3 contains multiple protein-interaction domains, including an SPN domain, ankyrin-repeat region, SH3 and PDZ domains, a proline-rich interaction region, and a C-terminal SAM domain, allowing it to organize receptor-associated scaffolds within the postsynaptic density [13]. These domain architectures place NaV1.2, ankyrin-B, and SHANK3 in complementary dendritic compartments: the dendritic shaft membrane–cytoskeleton interface and the receptor-rich postsynaptic spine scaffold.

Despite their molecular differences, *SCN2A*, *ANK2*, and *SHANK*-family genes converge functionally on a shared cellular problem: how neurons coordinate intrinsic excitability with synaptic drive to stabilize dendritic input–output transformations. Dendrites actively filter, amplify, and integrate synaptic input through voltage-gated conductances, receptor composition, spine architecture, and cytoskeleton-linked scaffolding. Disruption of any of these layers can alter dendritic gain—how strongly and reliably a given pattern of synaptic input is converted into dendritic and somatic electrical output—together with plasticity thresholds and circuit stability [10,11,13,14]. This dendritic scaffolding framework complements the excitation/inhibition imbalance hypothesis [15] by focusing on an upstream cellular mechanism: the integrity of dendritic channel localization, postsynaptic receptor organization, and cytoskeleton-linked scaffolding. Such upstream disruption could produce different downstream excitability profiles depending on the cell type, circuit context, and developmental stage [16,17,18,19].

Here, we use the term framework in a functional, not biochemical, sense. ANK2 and SCN2A proteins form a directly demonstrated dendritic scaffolding module in neocortical pyramidal neurons, whereas SHANK-family proteins organize a complementary postsynaptic scaffold that converges on related dendritic computations. Thus, the proposed *ANK2*–*SCN2A*–*SHANK*-family framework does not imply that these proteins form a single molecular complex. Instead, it refers to layered mechanisms that together regulate dendritic excitability, postsynaptic organization, and synaptic maturation. Section 2 reviews this cortical template in detail, whereas Section 3 evaluates whether related mechanisms may operate in subcortical circuits. The starting point for considering these three genes together is therefore not a shared protein complex but a shared dendritic computation. We begin from the single interaction that is experimentally established—the direct, dendrite-specific anchoring of NaV1.2 by ankyrin-B in neocortical pyramidal neurons [11]—and ask which other high-confidence autism-risk genes shape the same computation. SHANK-family proteins meet this criterion because they organize the excitatory synaptic input that the ANK2–SCN2A machinery integrates and amplifies. In this framing the ANK2–SCN2A module is the demonstrated anchor, and the SHANK scaffold is included not as a binding partner but as the organizer of the synaptic events on which dendritic integration operates. This framework is particularly relevant to autism because many autism-associated traits involve differences in how sensory, social, motivational, and action-related information is integrated across time and context. Social approach–avoidance and social preference depend in part on bidirectional prefrontal–amygdalar circuits that assign affective valence to social cues [20,21,22]. Restricted and repetitive behaviors, flexible action selection, sensory reactivity, attentional filtering, and sleep regulation depend on corticostriatal and thalamocortical loops [23,24,25,26]. We therefore focus on dendritic integration not as a generic cellular phenotype but as a candidate mechanism through which genetically distinct neurodevelopmental variants may alter the gain, timing, and state dependence of circuit operations relevant to autism-associated behavior.

**Figure 3:**
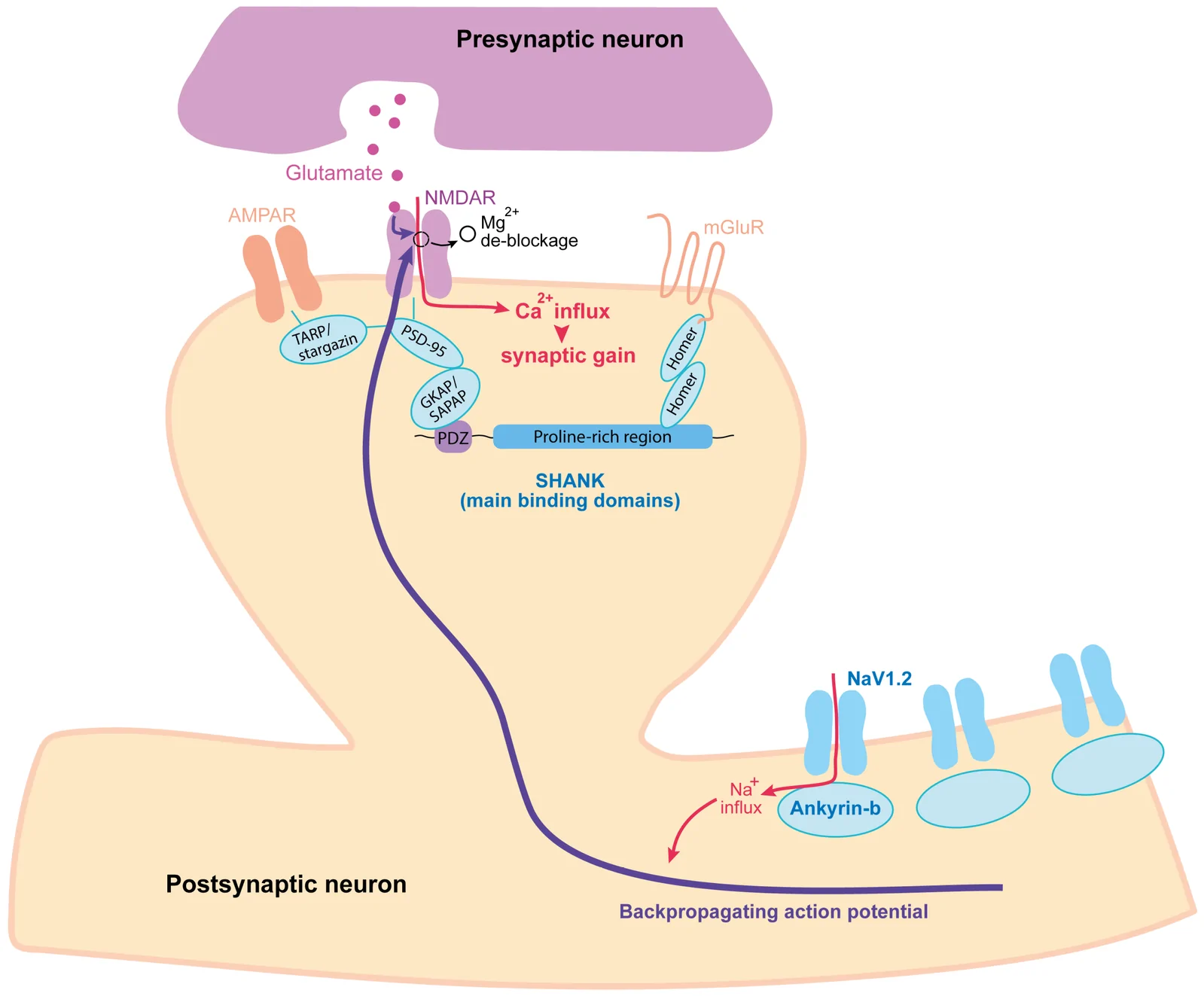
Proposed layered dendritic scaffolding framework at an excitatory postsynapse. In the dendritic spine, glutamatergic receptors (NMDA receptors, AMPA receptors, and group I metabotropic glutamate receptors) are organized within the postsynaptic density through indirect, layered linkages rather than direct binding to SHANK. NMDA receptors couple to PSD-95 and, via GKAP/SAPAP, engage SHANK-family proteins; AMPA receptors couple to PSD-95 through stargazin/TARPs; and group I metabotropic glutamate receptors couple to SHANK through Homer. Connecting thin lines indicate simplified molecular associations within larger receptor–scaffold and membrane–cytoskeleton networks; not all intermediary proteins are depicted. In the adjacent dendritic shaft, ankyrin-B (*ANK2*; 220 kDa isoform) anchors NaV1.2 (*SCN2A*) within the dendritic membrane-associated cytoskeletal scaffold, supporting dendritic sodium signaling, backpropagating action potentials, and active dendritic integration. These two modules are spatially adjacent and functionally convergent on dendritic input–output gain, but they do not represent a single biochemical complex. Abbreviations: AMPAR, AMPA receptor; GKAP/SAPAP, guanylate kinase-associated protein/SAP90/PSD-95-associated protein; mGluR, metabotropic glutamate receptor; NaV1.2, voltage-gated sodium channel 1.2; NMDAR, NMDA receptor; PSD-95, postsynaptic density protein 95; TARPs, transmembrane AMPA receptor regulatory proteins.

The proposed dendritic framework is summarized in Figure 3, which distinguishes the *ANK2*–*SCN2A* dendritic-shaft module from the *SHANK*-family postsynaptic scaffold at an excitatory postsynapse. This mechanism is proposed to operate within cortico-subcortical circuits linking the prefrontal cortex, striatum, thalamus, and amygdala. The striatum currently provides the strongest subcortical test case, whereas the thalamus and amygdala remain promising but less established extensions.

## 2. A Cortical Template for Layered Dendritic Scaffolding

The mechanistic evidence for the proposed framework remains strongest in the cortex. This section therefore defines the cortical template before considering whether related mechanisms extend to subcortical circuits. The key point is not that ANK2, SCN2A, and SHANK-family proteins form a single biochemical complex. Rather, the evidence supports two adjacent dendritic modules: an ANK2–SCN2A module that controls dendritic NaV1.2 localization in the shaft, and a SHANK-family module that organizes receptor-associated scaffolds in dendritic spines (Figure 3). These mechanisms operate through distinct molecular interactions but converge functionally on dendritic input–output gain.

### 2.1. The ANK2–SCN2A Module: Dendritic Channel Localization

NaV1.2, encoded by *SCN2A*, plays a developmentally dynamic role in neocortical pyramidal cells. NaV1.2, together with NaV1.6, is among the principal voltage-gated sodium channels of excitatory pyramidal neurons, in contrast to the NaV1.1 channels enriched in inhibitory interneurons [27]. Early in development, NaV1.2 is enriched at the axon initial segment, where it contributes to action potential initiation. After the first postnatal week, NaV1.2 becomes increasingly important in somatodendritic membranes, where it supports backpropagating action potentials—spikes initiated near the axon and soma that travel back into the dendrites, signaling recent output to active synapses and, by depolarizing dendritic spines, helping convert coincident synaptic input into long-term potentiation—as well as dendritic Na^+^ influx and active dendritic integration [10,11,17]. *Scn2a* haploinsufficiency reduced dendritic Na^+^ influx, weakened backpropagation, lowered the AMPA:NMDA ratio, increased the proportion of silent GluN2B-enriched synapses, and blocked long-term potentiation induction [10]. The AMPA:NMDA ratio and the proportion of silent synapses—immature contacts that carry GluN2B-rich NMDA currents but lack functional AMPA receptors—are standard readouts of excitatory synapse maturation, so these shifts indicate arrested postsynaptic maturation rather than a purely intrinsic excitability change [10]. These deficits were not merely developmental: restoration of *Scn2a* in adulthood rescued several phenotypes, indicating that mature dendritic function depends on ongoing NaV1.2 availability rather than solely on developmental channel expression [11].

Nelson and colleagues [11] identified ankyrin-B as the key scaffold that anchors dendritic NaV1.2. Using epitope-tagged NaV1.2, *Ank2* knockout-and-rescue experiments, and immunoprecipitation from the adult neocortex, they showed that ankyrin-B binds NaV1.2 through the canonical ankyrin-binding motif in the channel’s II–III intracellular loop. Ankyrin-B was required for dendritic, but not somatic, NaV1.2 localization. Ankyrin-G, the dominant axonal ankyrin described in related neuronal scaffolding studies [28,29], could not substitute for ankyrin-B at the dendritic membrane, although both ankyrins can scaffold somatic sodium channels. This finding established a direct physical and functional convergence between two high-confidence autism-risk genes within neocortical pyramidal cell dendrites. This mechanism requires isoform precision. *ANK2* encodes multiple ankyrin-B isoforms with compartment-specific functions. The dendritic NaV1.2 scaffolding mechanism has been linked most directly to the canonical 220 kDa ankyrin-B isoform, whereas autism-associated mutations affecting giant 440 kDa ankyrin-B have been associated with axonal branching and aberrant long-range connectivity [12,30].

These represent distinct cellular mechanisms—dendritic channel localization versus axonal wiring defects—that may both contribute to neurodevelopmental phenotypes but through different routes. Ankyrin-B membrane targeting also depends on S-palmitoylation, which promotes dendritic membrane localization and supports NaV1.2 positioning in dendrites [31]. Thus, dendritic NaV1.2 localization is an actively regulated scaffolding process, not a passive distribution of sodium channels.

### 2.2. The SHANK Scaffold: Postsynaptic Receptor Organization

SHANK-family proteins organize a complementary layer of dendritic architecture within the postsynaptic density. Rather than anchoring NaV1.2 directly, SHANK proteins scaffold receptor-associated signaling complexes in dendritic spines. N-methyl-D-aspartate (NMDA) receptors couple to PSD-95 and from there to GKAP/SAPAP, which binds the PDZ domain of SHANK; α-amino-3-hydroxy-5-methyl-4-isoxazolepropionic acid (AMPA) receptors couple to PSD-95 through stargazin/TARPs and are tethered to the same postsynaptic platform; and group I metabotropic glutamate receptors couple to SHANK through Homer [32,33,34,35]. SHANK proteins then connect these receptor-associated complexes to actin-regulatory machinery through cortactin and related signaling modules. Thus, the receptor scaffold depicted in Figure 3 should be interpreted as a layered postsynaptic organization, not as direct binding of all receptor classes to SHANK.

This layered architecture is important because disruption at the *SHANK* level can affect several receptor-associated pathways simultaneously. *SHANK3* loss altered spine morphology, weakened excitatory synaptic transmission, and impaired synaptic plasticity [23,36]. Combined *Shank1* and *Shank3* deletion further impairs developmental Akt signaling, underscoring the developmental role of SHANK-family scaffolds [37]. *SHANK3* haploinsufficiency has also been associated with Ih channelopathy in human neurons, suggesting one mechanism by which postsynaptic scaffold disruption may alter dendritic excitability, synaptic integration, and neuronal morphology [38]. These findings support the idea that SHANK-family disruption can alter dendritic computations through postsynaptic scaffold instability, even though it does not directly reproduce the ANK2–SCN2A channel-localization mechanism.

The *SHANK* module should also be interpreted in a region- and isoform-sensitive manner. *SHANK3* provides the strongest entry point for the striatal and cortico-limbic components of this framework because of its strong autism association, its robust effects on excitatory synaptic maturation, and its prominent roles in striatal and cortico-amygdalar circuits [13,23,24]. However, *SHANK3* should not be forced into every subcortical context. *SHANK2* may be particularly relevant in selected thalamic regions, and region-specific *SHANK* expression differences need to be considered when translating between rodent and human data [39]. Thus, the ANK2–SCN2A module may converge with different SHANK-family scaffolds depending on the cell type, developmental stage, species, and subcortical region.

### 2.3. Functional Convergence

The strongest interpretation is not that SHANK3 directly binds the ANK2–SCN2A complex but that SHANK-family disruption may converge functionally with ANK2–SCN2A dysfunction at the level of dendritic computation. Ankyrin-B organizes the electrogenic machinery of the dendritic shaft by positioning NaV1.2, whereas SHANK-family proteins organize receptor-rich postsynaptic machinery within dendritic spines (Figure 3). These systems operate through distinct molecular scaffolds, but both influence how dendrites transform synaptic input into electrical and biochemical output.

The key link between the two modules is that dendritic integration is not a property of either compartment alone; they converge on a single physical event at the spine. NaV1.2-dependent backpropagating action potentials and dendritic Na^+^ signals supplied by the ankyrin-B–anchored shaft provide the postsynaptic depolarization that, coincident with glutamate release, relieves the Mg^2+^ block of NMDA receptors and admits the Ca^2+^ that induces plasticity [10,11,14]. Because those NMDA receptors are positioned and stabilized within the SHANK-organized postsynaptic scaffold [32,33,34,35], the SHANK module is the structural site at which the electrical output of the ANK2–SCN2A module is read out as synaptic plasticity. The two modules are therefore not merely adjacent: one supplies a coincidence signal, the other positions the coincidence detector.

This coupling is visible in the loss-of-function phenotypes themselves. Disruption of the dendritic-shaft module does not remain confined to the shaft: Scn2a haploinsufficiency lowers the AMPA:NMDA ratio, increases the proportion of silent GluN2B-enriched synapses, and blocks long-term potentiation [10]—all changes in the postsynaptic compartment that the SHANK scaffold organizes. SHANK-family loss converges on the same readout from the opposite direction, weakening excitatory transmission, destabilizing spines, and impairing plasticity [23,36,38]. Because both modules ultimately set the strength and plasticity of the same excitatory synapses, their disruption is predicted to interact rather than simply co-occur, which is the rationale for the compound and sequential genetic-interaction experiments proposed in the Discussion. This distinction is central to the framework. The ANK2–SCN2A module primarily affects dendritic sodium-channel localization, backpropagating action potentials, dendritic Na^+^ influx, synaptic maturation, and plasticity thresholds. The SHANK-family scaffold primarily affects postsynaptic receptor organization, spine structure, glutamatergic signaling, and activity-dependent synaptic regulation. Loss of either system can therefore alter dendritic input–output gain, but the physiological direction of the effect may differ across cell types and circuits.

This interpretation also clarifies the relationship between the dendritic scaffolding framework and the excitation/inhibition imbalance hypothesis. The excitation/inhibition framework describes circuit-level output in terms of the balance between excitatory and inhibitory signaling [15,19]. The dendritic scaffolding framework operates one level upstream, asking how neurons position the electrogenic and receptor machinery that determines how synaptic inputs are integrated. A neuron with disrupted dendritic NaV1.2 localization or destabilized SHANK-dependent receptor scaffolds may appear either hypoexcitable or hyperexcitable depending on compensatory adaptations, local channel expression, and circuit state [16,17,18,19]. Therefore, the proposed convergence does not predict a single direction of excitability change. Instead, it predicts impaired stabilization of dendritic input–output gain. Together, these cortical mechanisms provide a mechanistic template for testing whether autism-risk genes converge on dendritic input–output transformations across cortico-subcortical circuits.

## 3. Subcortical Extensions of the Framework

The cortical-centric framing of ANK2–SCN2A convergence partly reflects an experimental bias toward layer 5 pyramidal neurons, which are highly tractable for dendritic electrophysiology and imaging. However, autism-associated behavioral domains depend on recurrent cortico-subcortical loops, and the genes considered here are expressed beyond the cortex. The critical question is therefore not whether *SCN2A*, *ANK2*, and *SHANK3* are expressed at the subcortical level but whether related molecular logic also shapes dendritic function in striatal, thalamic, and amygdalar neurons.

The current evidence is uneven. The striatum provides the strongest subcortical case because both NaV1.2 and SHANK3 have established functional relevance in medium spiny neurons and corticostriatal behavior. The thalamus provides an emerging case, driven mainly by SHANK-related channelopathy and thalamocortical dysfunction. The amygdala provides a plausible but more speculative extension, supported primarily by SHANK3 circuit data and by the dependence of basolateral amygdala neurons on dendritic integration of long-range excitatory inputs. In each region, the contribution of *ANK2* remains the least defined and therefore represents the critical experimental gap.

Table 2 summarizes the established and hypothesized roles of the two modules across the cortical template and the three subcortical regions considered here, together with the current strength of evidence for dendritic convergence in each.

**Figure 4:**
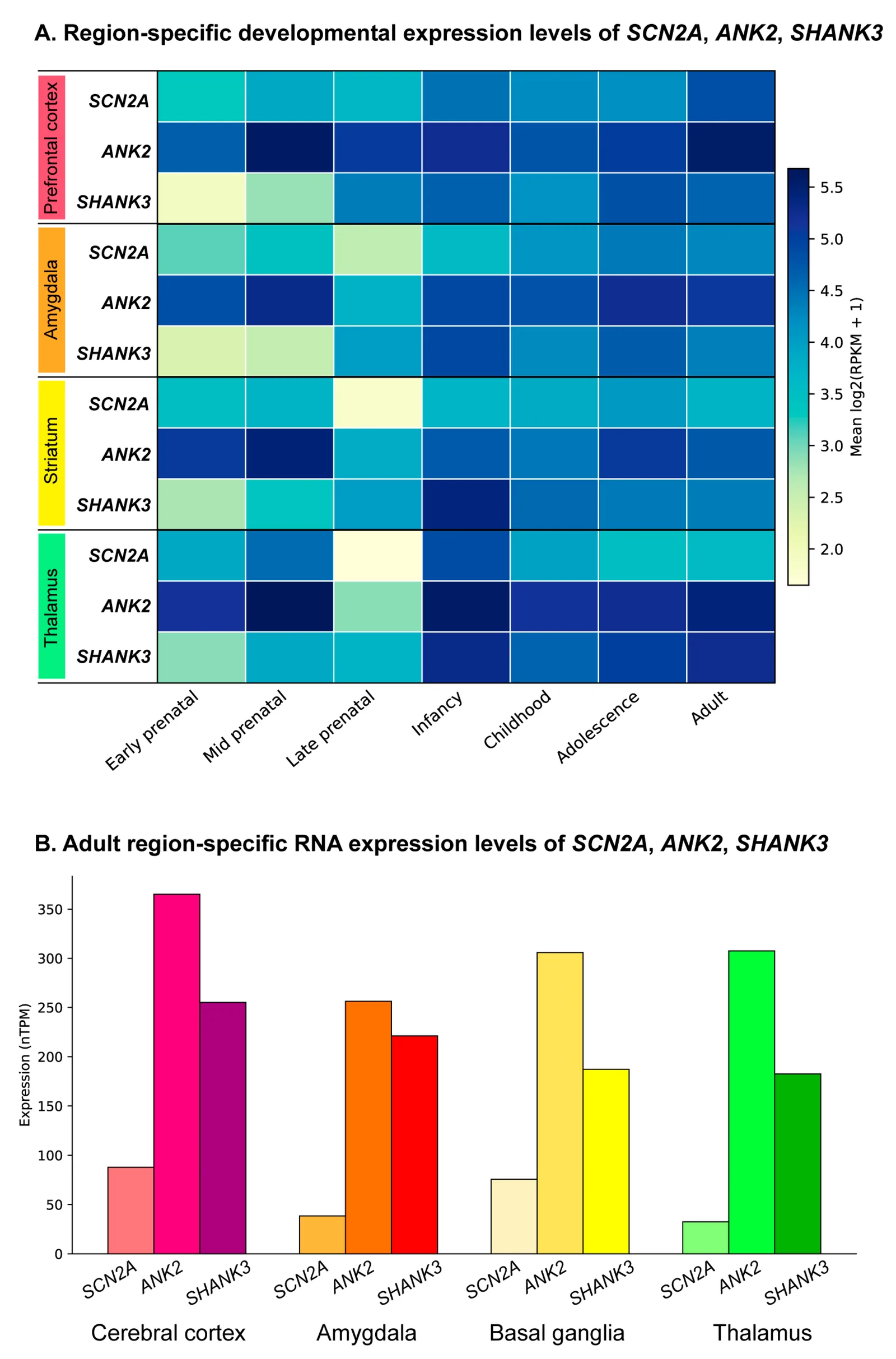
Regional and developmental expression of *SCN2A*, *ANK2*, and *SHANK3* in the human brain. (**A**) Developmental expression patterns from the BrainSpan Atlas of the Developing Human Brain. The heatmap shows *SCN2A*, *ANK2*, and *SHANK3* expression across prefrontal cortical regions, amygdala, striatum, and thalamic regions from prenatal development to adulthood. BrainSpan RNA-seq values were log2-transformed as log2(RPKM + 1) and averaged within each region–gene–stage category; the heatmap displays these mean log2(RPKM + 1) values. (**B**) Adult human brain-region RNA expression from the Human Protein Atlas. Grouped bars show normalized transcript expression (nTPM) for *SCN2A*, *ANK2*, and *SHANK3* across the HPA-defined regions cerebral cortex, amygdala, basal ganglia, and thalamus; these labels were retained as defined by the Human Protein Atlas and were not relabeled as more specific anatomical subdivisions. Together, these complementary datasets support the anatomical and developmental plausibility of the proposed framework while recognizing that transcript expression does not establish protein abundance, region-specific function, or molecular interaction.

We used the BrainSpan Atlas of the Developing Human Brain [51] to examine developmental expression trajectories across more anatomically specific regions. These data are summarized in Figure 4A. BrainSpan allowed us to group samples into prefrontal cortical regions, amygdala, striatum, and thalamic regions across developmental stages from prenatal development to adulthood. We log2-transformed the expression values as log2(RPKM + 1) and averaged them within each region–gene–stage category; the heatmap therefore displays mean log2(RPKM + 1) values across regions, genes, and developmental stages. These developmental data support the temporal plausibility of the framework by showing that *SCN2A*, *ANK2*, and *SHANK3* are expressed across relevant brain regions during developmental windows in which dendritic excitability, synaptic maturation, and cortico-subcortical circuit organization are established. However, as with the Human Protein Atlas analysis, these data provide expression context rather than direct evidence for *ANK2*–*SCN2A*–*SHANK* mechanistic convergence.

We also examined public human expression resources for *SCN2A*, *ANK2*, and *SHANK3*. First, we used the Human Protein Atlas brain RNA-seq dataset [52] to retrieve adult brain-region expression values for the three genes. These data are summarized in Figure 4B. To remain anatomically conservative, we retained the Human Protein Atlas region labels rather than relabeling broad anatomical categories as more specific subdivisions. Thus, Figure 4B shows *SCN2A*, *ANK2*, and *SHANK3* expression across the cerebral cortex, amygdala, basal ganglia, and thalamus. The basal ganglia category provides broad adult regional context for the striatal system, but it should not be interpreted as a striatum-specific measurement. These adult brain-region data support the anatomical plausibility that the genes are expressed in regions relevant to the proposed framework while underscoring that transcript expression alone does not establish protein abundance, dendritic localization, or molecular interaction.

The disease relevance of these genes is further reflected in their clinical and autism-genetic annotations (Table 3). All three genes are catalogued in OMIM with distinct Mendelian phenotype entries. *SCN2A* (MIM 182390) is associated with developmental and epileptic encephalopathy 11 (MIM 613721), benign familial infantile seizures 3 (MIM 607745), and episodic ataxia 9 (MIM 618924). ANK2 (MIM 106410) is associated with ankyrin-B–related cardiac arrhythmia and long QT syndrome 4 (MIM 600919). *SHANK3* (MIM 606230) is the principal driver of Phelan–McDermid syndrome, or 22q13.3 deletion syndrome (MIM 606232) [53]. In the SFARI Gene database, all three are classified as high-confidence autism-risk genes, with SHANK3 additionally designated as syndromic [54]. Their Eagle scores differ, reflecting differences in the current strength and replication of curated autism evidence across genes. This pattern is informative because the proposed convergence links genes whose Mendelian phenotypes are otherwise heterogeneous: an epilepsy/encephalopathy gene, a cardiac-arrhythmia gene with autism genetic evidence, and a syndromic neurodevelopmental gene. Thus, the framework does not imply a single clinical pathway. Instead, it proposes that genetically and clinically distinct risk genes may converge on a shared downstream cellular mechanism: dendritic input–output integration.

### 3.1. Striatum

The striatum is the most compelling subcortical site for testing an *ANK2*–*SCN2A*–*SHANK3* dendritic convergence model. NaV1.2 is strongly expressed in striatal medium spiny neurons as well as in medial prefrontal cortical pyramidal neurons [18,40]. Severe *Scn2a* deficiency unexpectedly elevated intrinsic excitability in striatal principal neurons through compensatory downregulation of KV1.1, an effect that was cell-autonomous, reversible by adult *Scn2a* re-expression, and likely relevant to corticostriatal circuit imbalance [18]. This finding is important because it shows that NaV1.2 loss does not produce a uniform reduction in excitability across all cell types. Its consequences depend on local channel compensation, developmental stage, and circuit identity. The paradoxical excitability increase in medium spiny neurons contrasts with the reduced dendritic Na^+^ influx observed in cortical pyramidal neurons [10], highlighting the cell-type specificity that any subcortical extension of this framework must accommodate.

*SHANK3* has particularly strong relevance in the striatum. It is the most abundant *SHANK* isoform in striatal circuits, and *Shank3* mutant mice showed autism-relevant behavioral phenotypes, including repetitive grooming and altered social interaction, alongside striatal synaptic and circuit alterations [23]. Global *Shank3* loss reduced Homer1b/c at the striatal postsynaptic density, altered mGluR5–Homer scaffolds, and disrupted corticostriatal circuit dynamics [24]. Region-specific manipulations further supported dissociable cortical and striatal contributions to autism-associated behavioral domains: striatal *Shank3* deletion was sufficient to elicit perseverative exploratory behavior, whereas broader forebrain excitatory deletion produced severe self-grooming phenotypes [42]. Early postnatal disruption of *Shank3* also accelerated corticostriatal maturation, producing precocious glutamatergic synaptogenesis and increased connectivity in medium spiny neurons [43,44]. These region-selective findings demonstrate that *SHANK3*-dependent postsynaptic scaffold disruption in the striatum is sufficient to produce autism-relevant behavioral changes that are partly independent of cortical effects. Together with human neuroimaging evidence linking caudate nucleus growth trajectories to restricted and repetitive behaviors [55], these findings position the striatum as a key site for linking dendritic scaffolding to repetitive behavior, behavioral flexibility, and reward-guided action selection.

By contrast, *ANK2* function in striatal dendrites remains poorly characterized. Ankyrin-B is broadly expressed across the central nervous system [12,41], and autism-associated *ANK2* mutations targeting giant 440 kDa ankyrin-B caused ectopic axonal branching and aberrant connectivity across cortical and subcortical tracts [30]. These findings support a broad role for ankyrin-B in neuronal wiring, but they do not yet demonstrate that the canonical 220 kDa ankyrin-B isoform scaffolds dendritic NaV1.2 in medium spiny neurons. This distinction is essential because medium spiny neurons are not simplified subcortical equivalents of cortical pyramidal cells. They differ profoundly in morphology, intrinsic conductances, dopamine modulation, corticostriatal and thalamostriatal input structure, and spine-based integration.

Therefore, the key prediction is not that *Scn2a*, *Ank2*, and *Shank3* mutations produce identical phenotypes across the cortex and striatum. Rather, the prediction is that they destabilize dendritic input–output transformations required for corticostriatal computation. Conditional *Ank2* deletion in Drd1- and Drd2-expressing lineages, combined with dendritic Na^+^ imaging and behavioral comparison across *Scn2a*, *Ank2*, and *Shank3* models, would directly test this hypothesis.

### 3.2. Thalamus

The thalamus has emerged as a central node in autism-relevant circuit models, particularly through its roles in sensory gating, sleep regulation, attentional control, and cortico-subcortical coordination [25,26]. However, the thalamus should not be treated as a homogeneous structure. Thalamocortical relay neurons, thalamic reticular nucleus neurons, first-order nuclei, and higher-order nuclei differ in intrinsic conductances, synaptic connectivity, behavioral roles, and state-dependent firing modes. Any subcortical dendritic convergence model must therefore be tested at the level of specific thalamic cell types and nuclei.

*SHANK*-family expression supports this need for regional precision. *SHANK3* is expressed in the human thalamus, while *SHANK2* shows particularly high neuropil density in selected thalamic regions [39,45]. These findings suggest that *SHANK*-dependent postsynaptic organization may contribute to thalamic function, but the relevant *SHANK* isoform may vary across nuclei and species. This distinction is especially important for translational research because *SHANK2* and *SHANK3* expression patterns may not map uniformly across rodent and human thalami [39]. Functional evidence already supports thalamic involvement in *Shank3*-related phenotypes. *Shank3* mutant mice displayed abnormal thalamocortical neuron excitability linked to HCN2 channel dysfunction in the ventral posteromedial nucleus, and correction of this channelopathy ameliorated sensory hypersensitivity and sleep fragmentation phenotypes [46]. Subtype-specific changes have also been reported in the thalamic reticular nucleus of *Shank3* mutant mice, including altered rebound burst firing in first-order projecting thalamic reticular neurons, more subtle changes in higher-order projecting thalamic reticular neurons, and reduced inhibitory drive to relay nuclei [47]. These findings align with broader models in which thalamic reticular dysfunction contributes to sensory and attentional phenotypes in neurodevelopmental conditions [25,26,48].

They are particularly relevant to autism-associated sensory reactivity, attentional filtering, and sleep–wake regulation, which depend on state-dependent thalamocortical and reticular gating. Whether *SCN2A* and *ANK2* participate in thalamic dendritic excitability remains unresolved. NaV1.2 has been reported in thalamic relay neurons [40], and both thalamocortical relay and thalamic reticular neurons rely on active dendritic conductances to shape burst firing, synaptic integration, and state-dependent gating. The cortical *ANK2*–*SCN2A* mechanism therefore provides a biologically plausible model, but direct evidence is still lacking. In particular, no study has yet demonstrated that ankyrin-B anchors NaV1.2 in thalamic dendrites or that *Ank2* haploinsufficiency phenocopies *Scn2a*-related thalamic dendritic defects. The key experiments would determine whether ankyrin-B and NaV1.2 colocalize in dendrites of thalamocortical relay and thalamic reticular neurons, whether *Ank2* loss reduces dendritic Na^+^ influx in these cells, and whether this interacts with *Shank*-dependent HCN, glutamatergic, or inhibitory synaptic alterations.

### 3.3. Amygdala

The amygdala provides a plausible but more speculative extension of the dendritic convergence model. SHANK proteins are expressed in amygdalar circuits, and SHANK3 has functional relevance in cortico-amygdalar pathways, although whether this intersects with *ANK2*–*SCN2A*-dependent dendritic excitability in basolateral amygdala neurons remains unknown [39,45,50]. Circuit-selective Shank3 deletion in the medial prefrontal cortex-to-basolateral amygdala projection produced altered dendritic structure, increased excitatory drive, reduced inhibitory drive, and altered sociability, paralleling altered functional connectivity of this circuit in autistic individuals [22]. This projection forms part of a broader prefrontal-to-basolateral amygdala pathway through which prefrontal cortical inputs differentially recruit distinct basolateral amygdala projection neuron populations [20] and through which amygdala-to-prefrontal projections guide behavior amid conflicting cues of reward and punishment [21]. Valence-encoding medial prefrontal cortex–basolateral amygdala subcircuits shape social approach–avoidance behavior, consistent with the view that dendritic disruption within this pathway could alter social-valence computation [16]. Together, these studies position social approach–avoidance and social preference as cortico-amygdalar behavioral readouts through which altered dendritic integration could be tested in autism-relevant models.

However, the roles of *SCN2A* and *ANK2* in amygdalar dendrites remain largely unexplored. Basolateral and lateral amygdala principal neurons integrate glutamatergic inputs from prefrontal, thalamic, hippocampal, and sensory association regions, including prefrontal-to-basolateral amygdala projections and direct thalamo-amygdala pathways that route sensory–affective information from posterior thalamic nuclei to the lateral amygdala. These pathways are sufficient, in parallel with thalamo-cortico-amygdala routes, to support associative affective learning [20,49]. Because these computations depend on dendritic excitability and synaptic integration, the *ANK2*–*SCN2A*–*SHANK*-family framework offers a plausible mechanistic hypothesis. Yet the evidence currently supports only the SHANK3 arm directly. Future translational studies should test whether NaV1.2 contributes to dendritic electrogenesis in basolateral amygdala principal neurons, whether ankyrin-B anchors NaV1.2 or other voltage-gated channels in these dendrites, and whether Shank3 disruption modifies related dendritic computations through postsynaptic scaffold instability.

## 4. Discussion

This review proposes that cortical *ANK2*–*SCN2A* convergence provides a mechanistic template for investigating autism-relevant alterations in dendritic integration across subcortical circuits. The framework does not claim that SCN2A, ANK2, and SHANK-family proteins form a single biochemical complex. Instead, it argues that dendritic channel localization, postsynaptic scaffold organization, and synaptic maturation represent layered mechanisms that can converge functionally on dendritic input–output gain. The Human Protein Atlas and BrainSpan analyses added here support the anatomical and developmental plausibility of this framework by showing expression of *SCN2A*, *ANK2*, and *SHANK3* across relevant human brain regions and developmental windows. However, these expression data should be interpreted as contextual support rather than direct evidence for protein abundance, dendritic localization, or molecular interaction.

### 4.1. Functional Convergence Predicts Destabilized Dendritic Gain Rather than a Fixed Excitability Shift

A major challenge for this framework is that dendritic convergence is unlikely to produce a uniform physiological signature across cell types and brain regions. SCN2A loss can reduce dendritic Na^+^ influx and weaken backpropagation in cortical pyramidal neurons, but it can also produce paradoxical hyperexcitability through compensatory changes in other conductances, as demonstrated in striatal medium spiny neurons [17,18]. Similarly, *SHANK3* loss can weaken excitatory synaptic maturation in some contexts while producing circuit hyperactivity or precocious connectivity in others [24,43]. Therefore, the relevant convergent phenotype may not be a fixed increase or decrease in excitability. Instead, it may be a failure to stabilize dendritic input–output gain across development, cell type, and behavioral state.

This interpretation clarifies the relationship between the dendritic scaffolding framework and the excitation/inhibition imbalance hypothesis [15,19]. The excitation/inhibition hypothesis describes circuit-level output in terms of the balance between excitatory and inhibitory signaling, often with emphasis on increased excitation. The dendritic scaffolding framework is not incompatible with this view, but it operates at a different explanatory level. Rather than describing the net output of a circuit, it addresses the upstream molecular mechanisms that determine how individual neurons position electrogenic and receptor-associated machinery within dendrites. A neuron with disrupted dendritic NaV1.2 localization or destabilized SHANK-dependent receptor scaffolds may appear either hypoexcitable or hyperexcitable depending on compensatory adaptations, local channel expression, synaptic input structure, and circuit state [17,18]. Thus, the same upstream scaffolding alteration could produce cell-type-specific downstream excitability changes. This cell-type specificity is reinforced by the division of labor within the sodium-channel family itself: NaV1.2, together with NaV1.6, predominates in excitatory pyramidal neurons [27], whereas NaV1.1 sustains high-frequency firing in inhibitory interneurons [56], and dendritic NaV1.2 can even drive dendritic gamma-aminobutyric acid (GABA) release in specialized neurons such as olfactory-bulb granule cells [57]—so the same loss-of-function variant may be read out differently depending on the channels and scaffolds a given neuron expresses. This framework therefore does not predict a uniform shift toward excitation or inhibition but rather a reduced capacity to maintain stable dendritic gain in a context-dependent manner.

### 4.2. Translational Window for Autism-Relevant Trait Assessment

In this view, dendritic scaffolding does not map into a single autism-associated trait. Rather, it may influence how distributed cortico-subcortical circuits regulate sensory reactivity, behavioral flexibility, sleep–wake state, and social-valence processing across development. This framing helps connect molecular mechanisms with behavioral domains without reducing autism to a single pathway or assuming that the same cellular alteration produces identical outcomes across circuits. It also explains why the striatum, thalamus, and amygdala differ in evidential strength: the striatum currently provides the clearest subcortical test case, whereas thalamic and amygdalar extensions remain more dependent on *SHANK*-related evidence and require direct tests of *ANK2*–*SCN2A* involvement.

**Figure 5:**
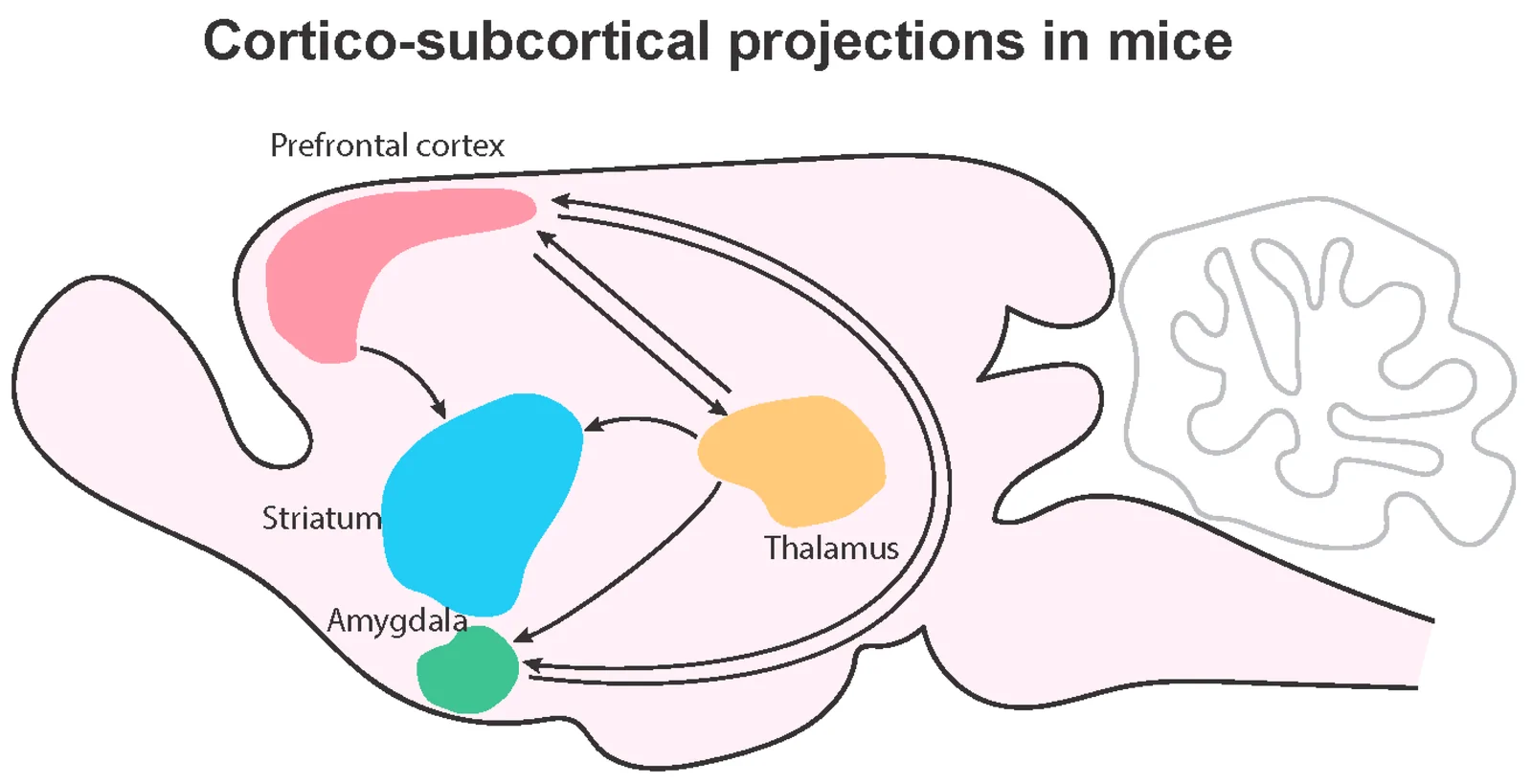
Cortico-subcortical projections relevant to the *ANK2*–*SCN2A*–*SHANK* framework. Schematic sagittal representation of cortico-subcortical projections in the mouse brain linking the prefrontal cortex (PFC), striatum, thalamus, and amygdala. Arrows depict the major projections relevant to this review: corticostriatal projections (PFC → striatum), thalamocortical and corticothalamic projections (thalamus ↔ PFC), thalamostriatal projections (thalamus → striatum), reciprocal prefrontal–amygdalar circuits (PFC ↔ BLA), and direct thalamo-amygdalar inputs (thalamus → amygdala). For simplicity, the schematic does not display polysynaptic return paths from the striatum to the cortex via the pallidum and thalamus. Abbreviations: BLA, basolateral amygdala; PFC, prefrontal cortex.

The cortico-subcortical circuitry that frames this proposal is defined largely in the mouse (Figure 5), so species translation requires caution. *SHANK*-family expression differs across species and subcortical regions; therefore, predictions derived from mouse models should be validated against human expression, post-mortem, and cell-type-resolved datasets wherever possible [39]. This point is especially important for the thalamus and amygdala, where *SHANK2* and *SHANK3* expression patterns may not map uniformly across rodents and humans. Conditional genetic models in mice, while powerful, may also differ from the gene dosage and genetic contexts observed in people. Gene-dosage sensitivity, genetic background effects, developmental compensation, and cell-type-specific isoform expression all represent potential confounds that must be considered when interpreting subcortical phenotypes in animal models.

### 4.3. Limitations

While this framework builds on converging genetic and physiological evidence linking SCN2A, ANK2, and SHANK-family genes to dendritic integration and circuit-level input–output gain, several limitations should be acknowledged. First, the proposed convergence is functional rather than biochemical: only the ANK2–SCN2A dendritic interaction has been directly demonstrated, in neocortical pyramidal neurons [11], whereas the inclusion of SHANK-family proteins rests on shared dendritic computations rather than on a shared molecular complex. Second, the human expression analyses presented here (Figure 4A,B) provide anatomical and developmental plausibility but do not establish protein abundance, dendritic localization, isoform usage, or mechanistic interaction in any subcortical region. Third, the subcortical extension of the ANK2–SCN2A module remains experimentally untested: there is currently no direct evidence that ankyrin-B anchors NaV1.2 in the dendrites of striatal medium spiny neurons, thalamocortical relay or reticular neurons, or basolateral amygdala principal neurons, and the framework should therefore be treated as a hypothesis to be tested rather than as an established mechanism. Fourth, the thalamic and amygdalar arms of the framework are supported almost exclusively by SHANK-related circuit and channelopathy data [39,45,46,47,48,50], so the strength of evidence is asymmetric across the three subcortical regions considered (Table 2). Fifth, much of the mechanistic evidence is derived from mouse models, and species- and isoform-level differences in SHANK and ankyrin-B biology [12,30,39] limit direct translation to human subcortical circuits. Sixth, this is a conceptual review rather than a systematic synthesis; literature selection was guided by the dendritic-integration logic of the framework, and the absence of formal meta-analytic procedures should be considered when weighing the evidence summarized here.

### 4.4. Experimental Priorities and Validation in Human Tissue

Several experimental priorities follow from this framework. Conditional *Ank2* deletion in striatal D1- and D2-expressing medium spiny neurons, thalamocortical relay neurons, thalamic reticular neurons, and basolateral amygdala principal neurons would test whether ankyrin-B supports dendritic excitability outside the cortex and whether its contribution differs across subcortical cell types. Dendritic Na^+^ and Ca^2+^ imaging across *Scn2a*, *Ank2*, and *Shank3* animal models would determine whether these genes produce convergent or cell-type-specific dendritic phenotypes. Region- and cell-type-specific rescue experiments would test whether restoring ANK2–SCN2A-dependent channel localization or SHANK-dependent postsynaptic organization in adulthood can normalize subcortical circuit function as adult rescue has already shown promise in *Scn2a* models [11,18]. Comparing the directionality of excitability changes across the cortex, striatum, thalamus, and amygdala would also be essential because NaV1.2 loss can produce either reduced or paradoxically increased excitability depending on cell type and compensatory channel regulation [17,18]. Finally, genetic-interaction designs, including compound or sequentially perturbed *Scn2a;Shank3* and *Ank2;Shank3* animal models, would test whether these modules interact at the level of dendritic gain rather than merely producing similar single-gene phenotypes.

Validation in human tissue should be treated as a priority rather than a caveat. Proximity-ligation and co-localization assays for ankyrin-B and NaV1.2 in human subcortical neurons, together with cell-type-resolved post-mortem and induced pluripotent stem cell-derived datasets, would help determine whether the dendritic scaffolding logic defined in the rodent cortex generalizes to the human striatum, thalamus, and amygdala. Such validation would be critical for establishing whether the *ANK2*–*SCN2A*–*SHANK*-family framework represents a shared point of convergence through which genetically distinct autism-associated variants alter cortico-subcortical circuit function.

## 5. Conclusions

SCN2A and ANK2 proteins converge directly in neocortical pyramidal cell dendrites, where ankyrin-B serves as an obligate scaffold for NaV1.2 and supports dendritic Na^+^ signaling, backpropagation, synaptic maturation, and plasticity. This module depends on isoform-specific ankyrin-B function and lipid-dependent membrane targeting. SHANK3 does not form a direct biochemical complex with ANK2–SCN2A but organizes a complementary postsynaptic scaffold that controls glutamatergic receptor complexes and synaptic plasticity through a layered PSD-95/GKAP/Homer architecture. Together, these systems define layered dendritic machinery required for stable excitatory integration.

The central unresolved question is whether this dendritic logic extends beyond the cortex. Current evidence makes the striatum the strongest subcortical test case because SCN2A and SHANK3 proteins both have established relevance in medium spiny neurons and corticostriatal behavior. The thalamus and amygdala provide compelling but more speculative extensions, supported mainly by *SHANK*-related channelopathy, synaptic dysfunction, and circuit phenotypes, while the contributions of *ANK2* and *SCN2A* remain largely undefined. Testing this framework across these circuits—and relating findings to human neuroimaging and behavioral stratification—could reveal whether dendritic scaffolding represents a circuit-selective point of convergence across genetically distinct neurodevelopmental conditions with autism-relevant phenotypes.

## Figures and Tables

**Table 1:** Selected Gene Ontology annotations for *SCN2A*, *ANK2*, and *SHANK3*.

Gene	Encoded Protein	GO Aspect	Selected GO Term	GO ID	QuickGO Qualifier	Relevance to the Framework
*SCN2A*	NaV1.2	Molecular function	Voltage-gated sodium channel activity	GO:0005248	enables	Supports the role of NaV1.2 in neuronal excitability and sodium-dependent dendritic signaling.
*SCN2A*	NaV1.2	Biological process	Sodium ion transmembrane transport	GO:0035725	involved_in	Links *SCN2A* to sodium flux across the membrane, consistent with active dendritic and action-potential-related signaling.
*ANK2*	Ankyrin-B	Molecular function	Structural constituent of cytoskeleton	GO:0005200	enables	Supports the role of ankyrin-B as a cytoskeleton-associated scaffold.
*ANK2*	Ankyrin-B	Molecular function	Spectrin binding	GO:0030507	enables	Links ankyrin-B to the spectrin-based cytoskeleton.
*ANK2*	Ankyrin-B	Molecular function	Transmembrane transporter binding	GO:0044325	enables	Supports the role of ankyrin-B in coupling membrane proteins, including channel-associated proteins, to cytoskeletal scaffolds.
*SHANK3*	SHANK3	Cellular component	Postsynaptic density	GO:0014069	located_in/is_active_in	Places SHANK3 within the excitatory postsynaptic scaffold.
*SHANK3*	SHANK3	Biological process	Synapse assembly	GO:0007416	involved_in	Supports the role of SHANK3 in synaptic organization and maturation.
*SHANK3*	SHANK3	Biological process	Regulation of synaptic plasticity	GO:0048167	involved_in	Links SHANK3 to activity-dependent synaptic regulation.

Selected human Gene Ontology (GO) annotations were retrieved from QuickGO and organized according to the three GO aspects: molecular function, cellular component, and biological process. Evidence codes: IBA, inferred from biological aspect of ancestor; IDA, inferred from direct assay; IEA, inferred from electronic annotation; IMP, inferred from mutant phenotype; IPI, inferred from physical interaction; ISS, inferred from sequence or structural similarity.

**Table 2:** Roles of the *ANK2*–*SCN2A* (NaV1.2–ankyrin-B) module and the *SHANK* family across the cortical template and subcortical circuits.

Region	NaV1.2–Ankyrin-B Module (*ANK2*–*SCN2A*)	*SHANK* Family	Status of Dendritic Convergence
Neocortex (PFC)	Demonstrated: ankyrin-B directly anchors dendritic NaV1.2; supports backpropagation, dendritic Na^+^ influx, the AMPA:NMDA ratio, and LTP; adult rescue restores function [10,11,17].	Organizes the excitatory postsynaptic density; loss alters spine morphology, excitatory transmission, and plasticity [13,23,36,38].	Established cortical template; the only region with a demonstrated ANK2–SCN2A dendritic complex.
Striatum	NaV1.2 expressed in medium spiny neurons; severe *Scn2a* loss paradoxically raises medium spiny neuron excitability via KV1.1 downregulation, reversible by adult rescue [18,40]; ankyrin-B is broadly expressed but its scaffolding of dendritic NaV1.2 in medium spiny neurons is untested [12,30,41].	*SHANK3* is the dominant striatal isoform; mutants show repetitive/social phenotypes with striatal synaptic and circuit changes; region-specific deletion drives perseveration; early loss accelerates corticostriatal maturation [23,24,42,43,44].	Strongest subcortical test case; the ANK2–SCN2A dendritic role is the key open question.
Thalamus	NaV1.2 reported in relay neurons; no evidence that ankyrin-B anchors NaV1.2 in thalamic dendrites [40].	*SHANK3* expressed (*SHANK2* enriched in some nuclei); *Shank3* mutants show thalamocortical HCN2 channelopathy (sensory hypersensitivity, sleep fragmentation) and reticular burst-firing changes [39,45,46,47,48].	Emerging; carried by SHANK-related channelopathy and circuit data; ANK2–SCN2A untested.
Amygdala	Roles of SCN2A and ANK2 proteins in amygdalar dendrites largely unexplored; no direct evidence.	*SHANK* expressed; circuit-selective *Shank3* deletion in the mPFC→BLA projection alters dendritic structure, excitatory/inhibitory balance, and sociability within valence circuits [16,20,21,22,39,49,50].	Speculative; supported only through the SHANK arm.

Citation numbers refer to the reference list. The “NaV1.2–ankyrin-B module” denotes the experimentally demonstrated ANK2–SCN2A dendritic complex; the “SHANK family” denotes the postsynaptic scaffold organized by SHANK proteins, principally SHANK3; PFC, prefrontal cortex; BLA, basolateral amygdala.

**Table 3:** Clinical (OMIM) and autism-genetic (SFARI Gene) annotations for *SCN2A*, *ANK2*, and *SHANK3*.

Gene	Gene (OMIM)	Associated OMIM Phenotype(s) [MIM]	SFARI Genetic Score	SFARI Eagle Score
*SCN2A*	182390	Developmental and epileptic encephalopathy 11 [613721]; benign familial infantile seizures 3 [607745]; episodic ataxia 9 [618924]	1 (high confidence)	109.3
*ANK2*	106410	Cardiac arrhythmia, ankyrin-B–related/long QT syndrome 4 [600919]	1 (high confidence)	10.8
*SHANK3*	606230	Phelan–McDermid syndrome (22q13.3 deletion) [606232]	1 (high confidence), syndromic	74.85

OMIM (www.omim.org); SFARI Gene 2026 Q1 release (www.gene.sfari.org). SFARI Gene score 1 denotes a high-confidence autism-risk gene; “syndromic” indicates association with a syndrome in which a substantial subpopulation develops autism. Eagle scores summarize the strength of curated autism evidence and should be interpreted as evidence-strength metrics rather than expression or effect-size measures.

## Data Availability

No new data were created or analyzed in this study. Data sharing is not applicable to this article.
